# Unusual Course of Scimitar Syndrome Preceded by Lung Hypoplasia

**DOI:** 10.1155/2019/8927243

**Published:** 2019-12-20

**Authors:** Yusuke Hoshino, Junichi Arai

**Affiliations:** Department of Neonatology, Ibaraki Children's Hospital, 3-3-1 Futabadai, Mito-city, Ibaraki 311-4145, Japan

## Abstract

In patients with Scimitar syndrome, right pulmonary artery hypoplasia is considered to lead to right lung hypoplasia because of decrease in blood flow. However, there are no reports wherein the change was actually detected. Thus, the exact developmental mechanism of right pulmonary artery hypoplasia and right lung hypoplasia in patients with Scimitar syndrome is unclear. We experienced a case of Scimitar syndrome preceding right lung hypoplasia, and right pulmonary artery hypoplasia gradually revealed with time. We hypothesized that, in our patient, the lung hypoplasia led to pulmonary artery hypoplasia due to decrease in blood flow. If there are no differences in the diameter of the left and right pulmonary artery in patients with Scimitar syndrome at birth, we propose the necessity of careful observation due to the possibility that pulmonary artery hypoplasia may develop in the future.

## 1. Introduction

Scimitar syndrome is a rare disease characterized by partial anomalous pulmonary venous drainage from the right lung into the inferior vena cava, hypoplasia of the right lung, and dextroposition of the heart. Patients with Scimitar syndrome have varied clinical presentations and are often diagnosed during adulthood because of the absence of symptoms. The exact developmental mechanism of right pulmonary artery hypoplasia and right lung hypoplasia in Scimitar syndrome is unclear [1]. Moreover, previous reports have not fully discussed the association between Scimitar syndrome and hemodynamic changes after birth. Herein, we report a case of Scimitar syndrome wherein differences were noted between the left and right pulmonary artery diameters after birth. Since this patient was admitted due to premature birth and regularly received chest radiography and cardiac ultrasonography during hospitalization, we were able to observe the changes associated with Scimitar syndrome by chance. We hypothesized that the disease follows a novel course based on hydrodynamic characteristics that explain how right pulmonary artery hypoplasia developed due to right lung hypoplasia.

Also, we obtained consent from the patient's parents with oral and written agreement.

## 2. Case Presentation

A male premature newborn infant was born via vaginal delivery at 31 weeks of gestation with a birth weight of 1,532 g. Antenatal ultrasound findings were normal. After birth, he experienced respiratory failure and required respiratory support with intubation. He was diagnosed with neonatal respiratory distress syndrome and treated with surfactant administration. In radiography, the right lung was less radiolucent than the left lung, and mesocardia was noticed; he was accordingly diagnosed with right lung hypoplasia. There were no differences between the left and right pulmonary artery diameters at that time.

**Figure 1:**
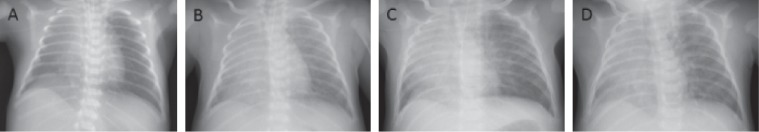
(a) Chest radiography image obtained at 1 day of life showing right lung hypoplasia and mesocardia. (b) Chest radiography image obtained at 14 days of life, showing that the heart had shifted slightly to the right side. (c) Chest radiography image obtained at 45 days of life showing that the heart had further shifted to the right side. (d) Chest radiography image obtained at 77 days of life (40 weeks postmenstrual age), the heart was completely located in the right thoracic cavity.

**Figure 2:**
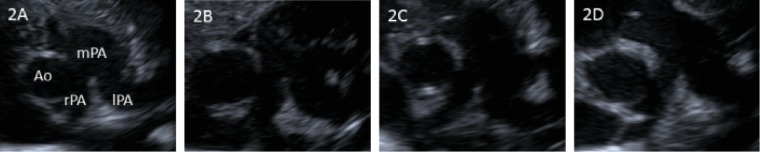
(a) No difference between the left and right pulmonary artery diameter noted at 1 day of life. (b) At 14 days of life, the right pulmonary artery (rPA) diameter was 3.0 mm, and the left pulmonary artery (lPA) diameter was 3.6 mm. (c) At 45 days of life, the rPA diameter was 3.1 mm, and the lPA diameter was 6.2 mm. (d) At 77 days of life (40 weeks postmenstrual age), the rPA diameter was 3.4 mm, and the lPA diameter was 6.8 mm.

**Figure 3:**
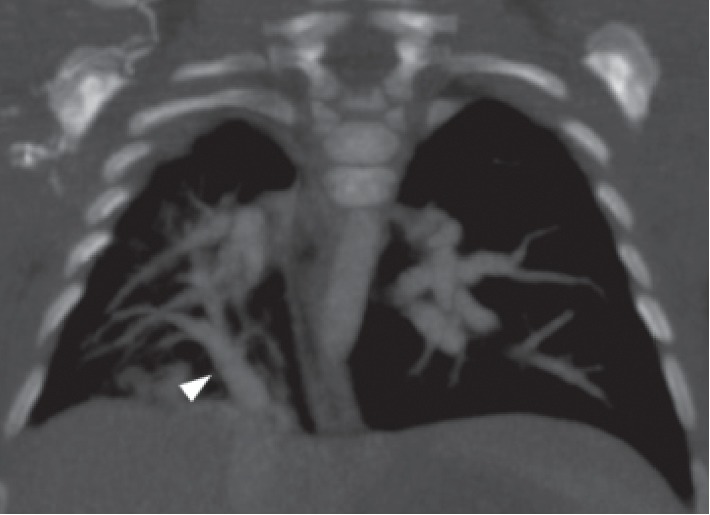
Contrast-enhanced computed tomography examination showed partial anomalous pulmonary venous drainage from the right lung into the inferior vena cava (arrow).

He regularly underwent chest radiography and cardiac ultrasonography during hospitalization. Serial examinations showed that the heart position had changed from the median position to the right position (Figure 1); the differences between the left and right pulmonary artery diameters gradually became apparent (Figure 2). At 77 days of life (40 weeks postmenstrual age), the right pulmonary artery diameter was 3.2 mm (−2.5 SD), whereas the left pulmonary artery diameter was 6.2 mm (+2.7 SD). Contrast-enhanced computed tomography examination showed partial anomalous pulmonary venous drainage from the right lung into the inferior vena cava at 73 days of life (Figure 3), and he was diagnosed with Scimitar syndrome due to right lung hypoplasia and dextroposition of the heart.

## 3. Discussion

Here, we report an unknown course of Scimitar syndrome. Previous reports suggested that right pulmonary artery hypoplasia leads to right lung hypoplasia because of decrease in blood flow [2, 3]. However, there have been no reports wherein the change has been actually detected. We hypothesized that lung hypoplasia led to right pulmonary artery hypoplasia associated with Scimitar syndrome after birth.

In our case, right lung hypoplasia occurred first during the fetal period. However, there were no differences between the left and right pulmonary artery diameters at birth (31 weeks of gestation). Shear stress in the tangential direction due to blood flow and extension stretch in the vertical direction due to intravascular pressure are considered to be necessary for blood vessel maturation [4, 5]. Since the pulmonary circulation had not been established during the fetal period, we considered that there were no differences in maturation between the left and right pulmonary arteries. After pulmonary circulation was established, we considered right lung hypoplasia led to pulmonary artery hypoplasia because of decrease in blood flow.

There have been several reports in which Scimitar syndrome was diagnosed during the neonatal period [1, 6]. In these reports, right pulmonary artery hypoplasia did not occur at birth. However, there was a possibility of a future occurrence of pulmonary artery hypoplasia. If there are no differences between the left and right pulmonary artery in patients with Scimitar syndrome at birth, careful observation is necessary because of the possibility that pulmonary artery hypoplasia may develop after birth.

In a case of Scimitar syndrome increasing pulmonary blood flow or showing signs of right heart failure, surgical treatment is considered [7]. In our case, cardiac catheterization showed normal pulmonary-to-systemic blood flow ratio (Qp/Qs ratio = 1 : 1), and we diagnosed for mild pulmonary hypertension. Thus, our patient did not undergo surgical treatment and continues with regular follow-up.
